# Animal Models in Rheumatoid Arthritis: Is There a Correlation Between Autoantibodies in Human Pathology and Animal Models?

**DOI:** 10.3390/biology14050460

**Published:** 2025-04-24

**Authors:** Miguel Marco-Bonilla, Maria Fresnadillo, Macarena de la Riva-Bueno, Gabriel Herrero-Beaumont, Raquel Largo, Aránzazu Mediero

**Affiliations:** Joint and Bone Research Unit, Instituto de Investigación Sanitaria Fundación Jiménez Díaz, Universidad Autónoma de Madrid, 28040 Madrid, Spain; miguel.marcob@quironsalud.es (M.M.-B.); maria.fresnadillo@quironsalud.es (M.F.); macarena.riva.edu@quironsalud.es (M.d.l.R.-B.); gherrero@fjd.es (G.H.-B.); rlargo@fjd.es (R.L.)

**Keywords:** rheumatoid arthritis, autoantibodies, patients, animal models

## Abstract

RA is a chronic autoimmune disease characterized by synovial inflammation and joint damage, driven by autoantibodies such as ACPA, anti-CarP and RF. These autoantibodies, influenced by genetic and environmental factors, play a crucial role in RA pathogenesis through post-translational modifications like citrullination, carbamylation, and acetylation. The early detection of ACPA provides a potential window for intervention, while anti-CarP antibodies correlate with severe disease progression and RF aids in diagnosis. Translating these findings from human pathology to animal models presents significant challenges. Although the presence of adaptative immune cells (T cells) is well defined in animal models of RA, studies yield inconsistent results regarding autoantibody production and implication in the disease onset and progression, with varying detectability of ACPA, anti-CarP antibodies, and RF across different species and models. The collagen-induced arthritis (CIA) model shows PAD4 expression and citrullinated protein presence but inconsistent ACPA detection, while the K/BxN model elucidates the pathogenicity of anti-GPI autoantibodies and implicates Fcγ receptors in disease processes. Therefore, further research is needed to bridge the gap between animal models and human RA pathology. Future studies should focus on developing more representative animal models, exploring pharmacological targets and pathways that involve the interplay between anti-inflammatory and autoimmune responses, and investigating the complex interplay between genetic predisposition, environmental triggers, and autoimmune mechanisms. This approach may lead to improved early diagnostic tools, targeted therapies, and potentially preventive strategies for RA, ultimately enhancing patient outcomes and quality of life.

## Simple Summary

Rheumatoid arthritis (RA) is a chronic autoimmune disease characterized by joint inflammation and destruction, driven by autoantibodies such as anti-citrullinated protein antibodies (ACPA), anti-carbamylated protein antibodies (anti-CarP), and rheumatoid factor (RF). The production of these antibodies is influenced by genetic and environmental factors, such as smoking and infections, and they play a key role in early disease detection and progression, often forming years before symptoms appear. Animal models of RA provide valuable insights into disease pathogenesis. However, the presence and contribution of autoantibodies in these models are inconsistent. While anti-CarP antibodies are reliably detected in several models, ACPA and RF show variable results depending on the species and methodologies used. Despite these limitations, animal models remain essential for exploring RA’s immune mechanisms and testing new treatments. This study highlights the need for improved animal models that better reflect human adaptative immunity in RA, thereby enhancing our understanding of autoimmune responses.

## 1. Introduction

Rheumatoid arthritis (RA) is a chronic, systemic autoimmune disease characterized by persistent inflammation of the synovial tissue [1]. This immune response involves the infiltration of T cells, B cells, and macrophages into the synovial membrane, leading to an abnormal reaction in synovial tissue-resident macrophages, which undergo hyperproliferation and contribute to cartilage and bone destruction [2,3,4].

The pathogenesis of RA is complex and not fully understood, but it is known that autoimmunity (developed by adaptative immune system) begins years before clinical symptoms appear, marked by the production of autoantibodies such as anti-citrullinated protein antibodies (ACPA), anti-carbamylated protein antibodies (anti-CarP) and rheumatoid factor (RF) [5]. The role of the innate immune system, synovial tissue-resident macrophages, is crucial in the early stages and progression of symptomatic RA, as they maintain the immune response and facilitate disease progression [6,7]. Additionally, genetic predisposition and environmental factors, such as smoking, contribute to the development of adaptative immune response in RA by influencing the production of autoantibodies in preclinical stages of the disease [8,9]. There are a large number of autoantibodies reported in RA patients [10].

Most of animal models of RA, develop many key features of the human disease that help in the study of disease mechanisms of innate immunity, histopathology in the synovial tissue and therapeutic interventions [11]. The main characteristic that develops these models is the synovial inflammation, characterized by hyperplasia of the synovial membrane, also known as pannus formation [12]. This invasive tissue is highly vascularized and infiltrated by a variety of immune cells, including macrophages and lymphocytes, which contribute to the perpetuation of local inflammation in the animal models [12]. Clinically, this manifests as swelling and redness in affected joints, especially in the paws, and is often accompanied by pain and reduced mobility [12]. As the disease progresses in animals models of RA, cartilage degradation becomes evident, driven largely by the activity of matrix metalloproteinases (MMPs), which break down key components of the extracellular matrix [12]. Simultaneously, bone erosion occurs as a result of osteoclast activation, mediated by the receptor activator of nuclear factor kappa-B ligand (RANKL) pathway and pro-inflammatory cytokines [11,12]. Moreover, cytokine dysregulation is a central driver of pathology in these models. Elevated levels of tumor necrosis factor-alpha (TNF-α), interleukin-1 beta (IL-1β), interleukin-6 (IL-6), and interleukin-17 (IL-17) are consistently observed and contribute to joint destruction, systemic inflammation, and the development of joint deformities over time [11,12].

Together, these features make animal models a valuable platform for dissecting the molecular and cellular mechanisms underlying RA innate immunity [6]. Nevertheless, although our understanding of the role of autoantibodies and adaptative immunity in the pathogenesis of RA patients has advanced, the correlation with animal models remains a subject of ongoing debate and controversy [13]. The presence of helper T-cells and their role in the development of inflammatory process and joint destruction has been well described in animal models of RA [14]. However, the presence of adaptive immune system cells or development of inflammatory pathology in these models does not correlate with the presence or role of autoantibodies as it does in RA patients [12,15,16]. Consequently a critical gap persists in our understanding of autoantibody generation in animal models of RA [12]. This aspect remains insufficiently explored in the literature. In this review, we summarized current knowledge and limitations about autoantibody response and their association with RA pathology in animal models. In the absence of these critical considerations, fundamental immunological characteristics risk being overly simplified, which may hinder the effective translation of experimental findings into valuable therapeutic interventions.

## 2. Rheumatoid Factor in RA Patients

Rheumatoid factors (RF) are antibodies that target the Fc region of IgG molecules, and they can belong to various immunoglobulin isotypes, including IgM, IgA, IgD, IgG, and IgE [17,18] been the IgM isotype the most relevant in clinical diagnosis [17]. These antibodies are thought to have evolved in humans as a mechanism to aid in the removal of immune complexes from the circulatory system [18]. RFs were first described in 1940 by Waaler, who identified a factor with hemagglutinating activity in the serum of a patient with RA, a discovery later confirmed as RF by Pike in 1949 [19,20].

RF plays a significant role in the diagnosis of RA and was incorporated in 1987 by the American College of Rheumatology as a criteria for RA classification [21]. However, its role has been somewhat reconsidered, as RF is also present in other conditions such as Sjögren’s syndrome, systemic lupus erythematosus, chronic infections (e.g., syphilis, tuberculosis), liver diseases, and even in healthy individuals, particularly the elderly [22,23,24,25,26,27]. Also, RF is present in about 25–30% cases of monoclonal gammopathy of undetermined significance, smoldering multiple myeloma or multiple myeloma [28]. Despite these associations, RF remains important in RA, where it is often used in conjunction with ACPA, which have higher specificity for RA [13].

The sensitivity of RF in detecting RA ranges from 41% to 66% for early RA and 62% to 87% for established RA, with specificity ranging from 43% to 96%, though most studies report a specificity higher than 70% [29] (Table 1). Despite the growing prominence of ACPA in diagnosing RA due to its higher specificity, RF continues to be a valuable marker, often complementing ACPA in the clinical evaluation of RA [29]. Also, elevated levels of RF, particularly in combination with ACPA, are associated with high inflammatory cytokines secretion (such as TNFα, IL-6 and IL-1β) and higher likelihood of joint damage in RA patients [30,31]. Understanding the interplay between RF and other biomarkers continues to be a critical area of research, particularly as personalized treatment strategies emerge in the management of RA.

## 3. ACPA in RA Patients

ACPA are crucial indicators in diagnosing and understanding the development of RA [32] (Table 1). These antibodies recognizes citrullinated proteins, a post-translational protein modification [38]. This modification is facilitated by protein arginine deiminase (PAD) enzymes, which convert arginine into citrulline [39]. The history of ACPA research dates back to 1964 with the identification of anti-perinuclear factor, followed by the discovery of anti-keratin antibodies [32]. These antibodies were found to be highly specific for RA [32]. By 1998, van Venrooij’s group demonstrated that the common denominator in these autoantibody systems was the reactivity against citrullinated peptides [40]. This discovery expanded our understanding of the immune mechanisms underlying RA and led to the term ‘citrullinome,’ which refers to the entire complex of citrullinated proteins that can trigger an immune response in RA patients [41]. The identification of numerous citrullinated proteins, such as fibrinogen, vimentin, aldolase A, collagen type II and α-enolase, among others, explains the complexity of the autoimmune response in RA [10].

Humans possess five PAD isotypes (PAD1–4 and PAD6), each with distinct roles, distributions, and implications for health [39,42]. Hypercitrullination in RA results from increased PAD activity, leading to abnormal citrullination that triggers inflammation and contributes to disease development [43]. Protein denaturation may also occur [44]. During extensive cell death, PAD enzymes and citrullinated proteins are released into the extracellular medium, exceeding immune cells capacity to manage them, potentially triggering autoimmune diseases [43]. One example of this is the NETosis, a specific form of cell death characterized by the release of intracellular contents (such as DNA, histones, granular proteins, and cytoplasmic proteins), which results in the formation of a neutrophil extracellular trap (NET) [45]. Increased NETosis in RA provides a source of citrullinated autoantigens and PAD enzymes, which, upon release from intracellular compartments, can citrullinate extracellular proteins, further driving ACPA production [46].

Anti-citrullinated vimentin antibodies have been shown to strongly promote NET formation [45]. The presence of NETs further enhances the activity of synovial fibroblasts, which produce proinflammatory cytokines, chemokines, and increase the expression of adhesion molecules [45]. These proinflammatory cytokines, in turn, trigger additional NETs formation [45]. Moreover, ACPA-induced NETs formation may contribute to the ongoing inflammation and autoimmunization processes in RA [46].

**Figure 1:**
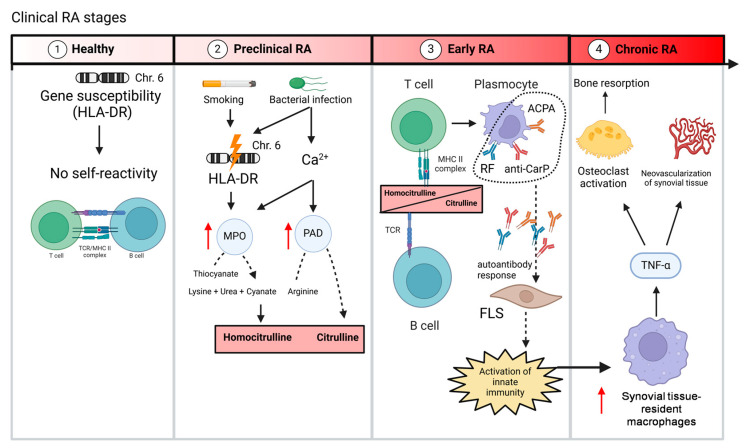
RA autoimmunity development in patients. Some healthy individuals have a genetic susceptibility at the HLA-DR1/3 locus that does not trigger an immune response in early years (1). In the preclinical RA phase, exposure to epigenetic factors such as smoking or bacterial infections generate alterations in the HLADR1/3 locus that led to increased PAD and MPO and thus to hypercitrullination and increased carbamylation. At the same time, the bacterial infection induces membranolytic damage through host and bacterial pore-forming proteins, which leads to high levels of calcium and an increase in citrullination (2). Carbamyl and citrulline peptides recognizes epitopes of B and T cells which interacts with plasmocyte to activate autoimmune response mediated mainly by ACPA, anti-CarP and RF (among many other autoantibodies like AAPAS, anti-GPI and anti-collagen II). The increase of systemic and synovial autoantibodies activates innate immunity (inflammasome) in synovial tissue-resident macrophages (3). Activation of innate immunity increases the response of macrophage which produces TNFα in the synovium. This molecule is responsible of bone resorption (mediated by osteoclast activation) and the neovascularization of synovial tissue (4). Red arrow indicates increase of enzyme activity in point 2 and increase of cell presence in point 4. Chr., chromosome; HLA-DR, human leukocyte antigen DR isotype; MHC II, major histocompatibility complex II; Ca^2+^, calcium; MPO, myeloperoxidase; PAD, protein arginine deiminase enzymes; TCR, T cell receptor; ACPA, anti-citrullinated protein antibodies; anti-CarP, anti-carbamylated protein antibodies; RF, rheumatoid factor; TNF, tumor necrosis factor.

The ACPA immune response unfolds in two phases [47]. The serum ACPA titer rises, and immune activity begins even before symptomatic RA appears [47]. ACPA can emerge decades before the clinical manifestation of RA, providing a window of opportunity for early intervention [30]. The first phase, known as the “first hit,” involves a breakdown in immune tolerance that leads to the production of low-level, low-activity ACPAs [47]. This phase is typically triggered by environmental factors or genetic predispositions associated with RA risk and can persist for many years without symptoms [48] (Figure 1). In the second phase, called the “second hit,” additional arthritic factors trigger a response where citrulline-specific B cells receive assistance from T cells, sparking an inflammatory autoimmune reaction [47]. As a result, ACPA levels raise, the diversity of antibodies increases, and the ACPA response matures [47] (Figure 1). Longitudinal studies of blood samples from individuals who later developed RA have shown that ACPA levels gradually increase over time, correlating with the progression of the disease [47].

In RA patients, ACPA can exist in various isotypes, including IgG, IgA, IgM, and even IgE [49]. Among these, IgG is the most prevalent in RA patients, with IgG1 and IgG4 being the most common subclasses [50]. One of the key ways that ACPA contributes to RA is through their interaction with immune cells, particularly macrophages [51]. ACPA can bind to Fc receptors (predominantly FcγRI and FcγRIIIA) on the surface of macrophages, triggering the activation of the complement system and the release of pro-inflammatory cytokines such as TNF-α, IL-1β, and IL-6 [52] (Figure 1). These cytokines are central to the inflammatory response in RA and play a crucial role in the disease’s pathology, like synovial neovascularization with ectopic lymphoid neogenesis [52,53] (Figure 1). Furthermore, ACPA may promote bone destruction by activating osteoclasts, the cells responsible for bone resorption [54] (Figure 1). This interaction between ACPA and osteoclasts contributes to the characteristic bone erosions seen in RA patients [54] (Figure 1). Recent studies show that ACPA-positive patients not only have a higher disease activity index, but also exhibit induced serological markers of cell death and metabolism unlike ACPA-negative patients [55]. Also, longitudinal data demonstrate distinct clinical profiles in the symptomatic pre-arthritis phase of ACPA-positive and ACPA-negative RA [56]. ACPA-negative patients experience initial symptoms in upper extremities, whereas ACPA-positive patients more frequently reported both upper and lower extremity involvement [56]. At the time of first clinical presentation with arthralgia, ACPA-positive individuals exhibited longer symptom duration and fewer tender joints compared to ACPA-negative patients [56]. Despite this delayed presentation, ACPA-positive patients progressed to clinical arthritis more rapidly following arthralgia onset [56]. These findings underscore fundamental differences in the early clinical trajectory of RA subtypes, supporting the concept that ACPA-positive and ACPA-negative RA follow distinct pathophysiological pathways [55,56].

ACPA role in the pathogenesis of RA is also influenced by genetic and environmental factors [57]. Specific genetic predispositions, such as alterations in human leukocyte antigen (HLA) and the presence of certain alleles, increase susceptibility to RA and enhance the production of ACPA [58] (Figure 1). HLA class I and III are involved in presentation of intracellular peptides and complement activation, respectively [59]. HLA class II molecules are found on the surface of antigen-presenting cells, such as macrophages, B cells, and dendritic cells. Their primary function is to present peptides to T-helper CD4+ cells, which triggers their activation [60]. HLA-DRB1 is the most characterized risk allele in RA, which is related with the induction of pathogenicity of ACPA in early RA [9]. Recent studies suggest that HLA-DRB1 risk alleles are associated with the development of N-glycosylation sites in ACPA-IgG; however, the exact mechanism behind this association remains unclear [61]. However, since HLA-DR molecules (encoded by the HLA-DRB1 gene) are essential for the activation of CD4+ T cells, it is probable that risk alleles of HLA-DRB1 contribute to somatic hypermutation of ACPA, thereby enhancing their pathogenic potential [62] (Figure 1). Curiously, RA heritability between ACPA-positive and ACPA-negative patients are the same, with values around 66–68% [63]. However, the presence of *HLA* shared epitope alleles is found in 18% of ACPA+ patients and only 2% of ACPA− patients [63]. Therefore, genetic predisposition plays an important role in ACPA-positive RA patients [63].

Others non-HLA susceptibility genes have been related with ACPA response in patients with less prevalence [64]. *PTPN22* gene polymorphism like C1858T missense single-nucleotide is associated higher ACPA-levels in RA patients [64]. Also, *PADI4* gene polymorphism is associated with ACPA-positive RA patients (especially in HLA-DRB1*04-positive) [65]. These findings suggest that RA pathogenesis is driven by a complex model of genetic variability, involving both HLA and non-HLA genes [57]. Identifying these genetic markers holds promise for improving early diagnosis and implement preventative strategies tailored to individual risk profiles [58,64,65].

Environmental or epigenetic factors, such as periodontal disease and smoking, can also contribute to the development of ACPA and the subsequent onset of RA [8]. Smoking, for example, has been shown to up-regulate HLA-DRB1 and activates PAD citrullination to form neoantigens in the lungs [66] (Figure 1). The presence of periodontitis, particularly infection by the bacterium *Porphyromonasgingivalis*, which produces PAD enzymes, has also been linked to increased ACPA production (by NETosis) in RA [67,68]. Bacterial pore-forming proteins, such as toxins, trigger significant calcium influx, cell lysis, and excessive activation of PADs (Figure 1). This likely contributes to the sustained hypercitrullination observed in both the RA joint and extra-articular sites where the disease begins [69] (Figure 1).

## 4. Anti-Car-P in RA Patients

Carbamylation is a process that develops in the human body under uremic or inflammatory conditions [70]. It involves the attachment of a carbamoyl group, which is related to cyanate, to proteins or peptides [70]. This can happen through both enzymatic and non enzymatic pathways [70]. The result of carbamylation is the formation of homocitrulline or α-carbamylated proteins [70]. While carbamylation is often considered an enzyme-independent process, it typically occurs in uremic conditions when urea dissolves in water, spontaneously forming cyanate that carbamylates proteins [70]. Inflammation can also lead to carbamylation by causing the oxidation of thiocyanate into cyanate, a reaction catalyzed by myeloperoxidase and hydrogen peroxide [68].

In RA, carbamylation produces homocitrulline, which is immunogenic and induces the production of anti-CarP antibodies [68]. These antibodies are present in RA patients, regardless of their ACPA status, and show cross-reactivity with ACPA [71]. Anti-CarP antibodies have a high specificity for RA diagnosis, and their presence in ACPA-negative or ACPA-positive RA patients correlates with more erosive joint manifestations in RA [72] (Table 1). This suggests that anti-CarP antibodies may be linked to aggressive disease progression and joint damage [72]. As in ACPA, NETs serve as a source of carbamylated autoantigens [73]. In fact, there is an association between anti–carbamylated NET autoantibodies and erosive joint disease by increase of synovial tissue-resident macrophages differentiation to osteoclasts (up-regulation of RANKL) and stimulation of osteoclast-mediated matrix resorption [73] (Figure 1). In RA patients autophagy is able to promoted protein carbamylation in synovial tissue-resident macrophages [74].

Anti-CarP antibodies have been reported in 3 different isotypes, including IgM, IgA, and IgG, and can undergo isotype switching [50]. This process explains the low avidity of anti-Car-P in comparison with ACPA IgG in RA patients [50].

As in ACPA, genetic alterations and environmental conditions can induce modifications of proteins by carbamylation. Several studies reported that carbamylation of vimentin is inducible by cigarette smoke exposure [75]. Similar to the role of HLA-DRB1 alleles in ACPA-positive RA, specific genetic factors such as HLA-DR1-3 have been associated with anti-CarP antibody-positive RA, particularly in ACPA-negative individuals [76] (Figure 1). Other pathologic conditions, such as uremic conditions or renal disease, in which the body experiences high levels of urea, can also promote carbamylation in RA patients [77,78].

## 5. Other Autoantibodies Detected in RA Patients

Acetylation is a type of post-translational modification that occurs through both co-translational and post-translational mechanisms [34]. It involves the attachment of an acetyl group, donated by acetyl-coenzyme A, to either the N-terminus of proteins or to lysine residues. This process, facilitated by various N-terminal and lysine acetyltransferases, is reversible and can significantly impact protein function [34]. Similar to carbamylation, acetylation alters the properties of proteins or peptides by modifying lysine residues, which can result in the formation of new epitopes that may trigger immune responses [68]. Acetylated proteins have been shown to provoke the production of anti-acetylated protein antibodies (AAPAs), a phenomenon with potential implications for autoimmune disorders such as RA [34,79]. In seronegative RA, acetylation of histones (independent of PAD) has been found to cross-react with ACPA [68]. This cross-reactivity suggests that acetylation may play a role in the autoimmune processes seen in RA [68] (Table 1). Furthermore, Kampstra et al. proposed that autoantibodies targeting PTMs, including ACPAs, anti-CarP antibodies, and AAPAs, may be linked by shared cross-reactivity with various post-translated autoantigens [80].

Other studies are focused on glucose-6-phosphate isomerase (G6PI) antigens and autoantibodies in RA. Schaller et al. showed that elevated levels of anti-G6PI antibodies in serum and synovial tissue of RA patients [81] with G6PI antigen levels increased in active phase of RA [35]. However, they also discovered that these antibodies were not exclusive to RA patients, as they were present in patients with other types of inflammatory arthritis (such as crystal-induced arthritis, seronegative spondyloarthropathies, traumatic arthritis, gonococcal arthritis, adenocarcinomatous arthritis, undifferentiated inflammatory polyarthritis, polymyalgia rheumatic and systemic lupus erythematosus) [82]. This led to the suggestion that immune-driven inflammatory arthritis triggers an increase in anti-G6PI antibodies and G6PI/anti-G6PI immune complexes, which may contribute to the release of pro-inflammatory cytokines and play a role in the progression of inflammatory arthritis [82]. The study conducted by Yang et al. found that the sensitivity and specificity of G6PI antigens in RA patients were higher (about 75.0% to 93.3%) [35,81] (Table 1).

Antibodies to type II collagen are also related to autoimmunity in early RA (27–82% of patients) [83] (Table 1). This immune response of antibodies to type II collagen is related to damage in the joint cartilage in RA pathology [84]. Others autoantibodies have been described in RA patients, such as anti-nuclear antibody (ANA) or anti-histone, but with less prevalence or present in others immune pathologies [10].

## 6. Autoantibodies in Animal Models of RA

Animal models are essential tools for studying RA pathology, comorbidities associated to this disease, and evaluating potential treatments [12,85,86,87]. These models help researchers understand the disease’s pathogenesis and test the efficacy of anti-arthritic drugs before clinical use [12]. Commonly used models of RA include collagen-induced arthritis (CIA), collagen antibody-induced arthritis (CAIA), adjuvant-induced arthritis (AIA), K/BxN serum-transfer, TNF transgenic (hTNFtg) and pristine-induced arthritis (PIA) which are used to predict drug efficacy and study disease mechanisms [11]. These models vary in their ability to replicate human RA’s complexity, and their selection depends on the specific research question and the aspect of RA being studied [11]. Cells of the adaptive immune system, such as T-cells, play a crucial role in the early development of disease in animal models of RA [14]. However, the implication of autoantibodies in the adaptative immune response remains unclear in all of these animal models of RA [12].

The role of ACPA is crucial in the development of autoimmunity, and its prevalence in early RA patients is well-established. [88]. However, studies reported that ACPA response and the generation of autoimmunity is controversial in RA models. (Table 2). In 2003 the study by Vossenaar et al. showed positive mRNA expression of PAD2 in healthy and CIA mice, and PAD4 in CIA mice. On the other hand, PAD1 and PAD3 are not expressed in synovial tissue [89]. Only PAD4 protein expression was detected in the synovium of CIA mice, together with positive citrullinated fibrin, but no antibodies against cyclic citrullinated peptide (CCP) were detected [89]. These authors argued that anti-CCP autoimmunity would take too long to develop in the CIA model and would not be feasible to detect [89]. Surprisingly, in the study of Kuhn group, anti-CCP antibodies were detected in serum of CIA mice by ELISA [88]. This peptide was detected early, days after immunization and weeks before the visible arthritis phenotype. In this study, antibodies produced in CIA mice were specific to citrullinated filaggrin and fibrinogen proteins, which were found at elevated levels in the serum of RA patients [88]. In the same line, ACPA response was reported in pre-boosting mice model of CIA (five to eight days before the onset of arthritis). The progression of inflammation in CIA model (both acute and chronic arthritis) produced an increase of ACPA responses. Increase of citrullinated proteins in joint tissue was reported in CIA mice in this study [90]. On the contrary, Stoop’s group demonstrated by ELISA that there was no evidence of ACPA in the serum of CIA, AIA and PIA in rats or hTNFtg and CAIA in mice compared to healthy controls, however the study did not show the inflammatory state of the animal models [15,16]. Absence of ACPA in serum of CIA mice model was corroborated by Kim group study [91]. Furthermore, anti-CCP antibody was not detected in serum of CIA rats on day 28 after collagen induction [92]. CIA induction in rhesus monkeys did not induce the presence of serum CCP2-citrullinated, fibrinogen-citrullinated or myelin basic protein-citrullinated in comparison with RA patients as positive control [93]. ACPA analysis has been less described in other animal models of RA. In AIA rat model at day 16 of induction, anti-CCP levels were increased in plasma [94]. Also, anti-CCP was increased in serum of CFA induced rats at day 21 [95]. In K/BxN mice model PAD4 activity and NET formation have been observed. The overall findings suggest that PAD4 is not essential in this model. However, is sure a potential role for PAD4 during the disease priming phase [96].

On the other hand, there is a consensus on the presence of carbamylated antibodies and the development of the adaptative immune response in animal models of RA (Table 2). These antibodies were incremented in serum of CIA, AIA and PIA rats models [16]. However, models without implication of adaptative immunity like hTNFtg and CAIA mice did not present anti-CarP response [16]. In a parallel study in CIA mice, anti-CarP response in serum (IgG1 and IgG2 isotope) already appeared before edema [15]. In CIA model in rhesus monkeys only anti-CarP antibodies were detectable [93]. Anti-CarP antibody levels rose over time, and, similarly to that described inhumans and mice, these autoantibodies can be detected prior to the clinical onset of the disease [93]. Abatacept inhibited the development of anti-CarP antibodies after immunization in rhesus monkey CIA model [93].

The involvement of RF in autoimmunity in RA models is not well understood (Table 2). Kim et al. study, which demonstrated the positive ACPA in CIA mice, confirmed and increase of RF IgM isotype [91]. On the contrary, X. Zhao group not detected differences in serum RF between CIA rats and healthy [92]. In the same line, in CIA model of rhesus monkeys RF-IgM was undetectable by ELISA [93]. In AIA rats, RF was detected in serum 30 days after CFA immunization [94]. Therefore, it is possible that RF was dependent of animal species employed, and the type of immunization in RA models.

The CIA and K/BxN models generate autoimmunity against collagen II and GPI, respectively [12,83,84] (Table 2). These autoantibodies have been observed in seropositive RA patients [10]. In CIA mice model, antibodies to type II collagen were reported by Kuhn et al. for IgM isotype at day 15 of RA induction [88]. Also, Piao et al. observed positive antibodies to type II collagen (isotype IgG1) in CIA mice at day 32 post-induction [99].

In the serum of the F1 offspring from a cross between KRN-transgenic C57BL/6 and NOD mice (K/BxN serum), IgG anti-GPI autoantibodies are detected [97] (Table 2). Most of these IgG anti-GPI autoantibodies belong to the IgG1 subclass [97]. However, IgG2 anti-GPI autoantibodies are also present in K/BxN serum [97]. Of the three activating IgG receptors in mice (FcgR), FcgRIIIA can bind to both IgG1 and IgG2, while FcgRI and FcgRIV interact exclusively with IgG2 [97] (Table 2). In K/BxN mice, B cell clones specific to anti-GPI were found in the spleen, while they were less common in other lymphoid tissues and the synovial fluid [98]. Combinations of anti-GPI mAbs isolated from K/BxN mice and injected in healthy mice were able to develop conditions similar to RA [98] (Table 2).

## 7. Conclusions

The pathogenesis of RA is marked by a multifaceted autoimmune response involving genetic predisposition, environmental factors, and the interplay of innate and adaptive immune systems. ACPA, anti-CarP, and RF are key autoantibodies that not only aid in diagnosing RA but also provide insights into its progression and severity.

Although the adaptive immune response in animal models is characterized by the presence of T cells, the detection of autoantibodies, such as ACPA, anti-CarP, and RF, in various animal models of rheumatoid arthritis (RA) has revealed considerable variability and complexity in both their presence and their role in disease progression. While the CIA mouse model shows evidence of PAD4 protein expression and citrullinated proteins, both systemically and in the synovium, studies yield conflicting results on the presence of ACPA. Some studies detected anti-CCP antibodies early in disease progression, while others failed to confirm their presence. This inconsistency highlights the challenge of using ACPA as a biomarker in animal models. Anti-CarP antibodies have been more consistently detected across models, including CIA in mice and rhesus monkeys, with their appearance often preceding clinical disease onset. This suggests their potential utility in understanding the adaptive immune response in RA. On the other hand, RF responses appear to depend on the species and immunization type, as demonstrated by varied detection in CIA mice, rats, and rhesus monkeys. The K/BxN mouse model provides insight into the pathogenic role of IgG anti-GPI autoantibodies, with IgG1 and IgG2 subclasses detected in serum. These autoantibodies interact with activating Fcγ receptors and are implicated in arthritis-like disease. Therefore, the use of RA animal models has been essential to elucidate the immunopathological mechanisms underlying RA, but differences in autoantibody responses across models underscore the need for careful interpretation of findings and their translation to human RA.

## 8. Future Directions

While considerable progress has been made in elucidating the immunopathological mechanisms of RA, significant challenges persist in translating findings from animal models to human disease. Future research should aim to refine existing models and develop novel approaches to better recapitulate the complexity of human autoantibodies role in RA.

First, the development of animal models that accurately mimic the human adaptive immune response is crucial. These models should not only incorporate the presence of immune cells, such as T cells, but also facilitate the production of autoantibodies, which are essential for understanding autoimmune response in RA patients. Current models often fail to reproduce the diversity and kinetics of autoantibody production observed in patients.

Second, there is a critical need for the standardization of methodologies used to detect autoantibodies in preclinical studies. Variability in assay sensitivity and specificity contributes to conflicting results across models. Harmonized protocols would facilitate cross-study comparisons and improve reproducibility.

Third, longitudinal investigations are warranted to delineate the temporal development of autoantibodies in relation to disease onset and progression. Understanding the preclinical dynamics of autoantibody emergencecould help identify early immunological events and therapeutic windows.

Fourth, the integration of genetic, environmental and epigenetic factors, such as polymorphism in HLA or non-HLA susceptibility genes, smoking, microbiome alterations, or microbial triggers like *Porphyromonasgingivalis*, may help reproduce early disease-initiating events. These modifications could provide insight into gene-environment interactions driving post-translational modifications and immune dysregulation.

Fifth, expanding the repertoire of autoantibodies investigated in animal models—including anti-CarP antibodies, AAPAs, anti-G6PI, and anti-type II collagen—may improve our understanding of disease heterogeneity and uncover new therapeutic targets.

Finally, autoantibody responses should be utilized as pharmacodynamic endpoints in preclinical therapeutic studies. Evaluating how candidate drugs modulate ACPA, anti-CarP, and RF levels will be critical in assessing their impact on the autoimmune component of RA and their potential for disease modification.

By addressing these future directions, researchers can enhance the translational fidelity of RA models and accelerate the development of early diagnostic markers and targeted therapies, ultimately improving outcomes for patients with RA.

## Figures and Tables

**Table 1:** Characteristics of key autoantibodies detected in RA patients. ACPA, anti-citrullinated protein antibodies; anti-CarP, anti-carbamylated protein antibodies; RF, rheumatoid factor; anti-G6PI, anti-glucose 6-phosphoisomerase antibodies; anti-CCP, anti-cyclic citrullinated peptide; AAPA, anti-acetylated protein antibodie; anti-CII, anti-collagen type II; RA, rheumatoid arthritis; Ig, immunoglobulin; IL, interleukin; TNF, tumor necrosis factor; ACR, American College of Rheumatology and EULAR, European League against Rheumatism.

	Main Autoantibodies Produced in RA					
	RF	ACPA	Anti-CarP	AAPA	Anti-G6PI	Anti-Type II Collagen
Specificity	For IgM-RF isotype the specificity was about 89.5% to 90.7% [29]. For IgA-RF isotype the specificity was about 90.8% to 92.0% [29].	Specificity was about 85 to 99% [32].	Specificity was about 89% [33].	Specificity was about 77% [34].	Specificity was about 93% [35].	Specificity was about 95% [36].
Sensitivity	For IgM-RF isotype the sensibility was about 62.1% to 64.6% [29]. For IgA-RF isotype the sensibility was about 48% to 50% [29].	Sensibility was about 60 to 80% [32]. CCP2 assays have higher sensitivity than CCP1 assays [32].	Sensibility was about 44% [33].	Sensibility was about 60% for patients with early RA and about 68% for long-term RA [34].	Sensibility was about 75% [35].	Sensibility was about 79% [36].
Pathogenic relevance	RF interacts with IgG antibodies to create immune complexes. These complexes tend to accumulate within the synovial joints, where they activate the complement system and initiate a cycle of chronic inflammation [29]. This promotes the infiltration of immune cells such as neutrophils and macrophages into the joint space. In turn, these cells release pro-inflammatory cytokines which intensify inflammation and contribute to the degradation of joint tissue and the development of synovitis [29].	ACPA recognize and bind to citrullinated proteins leading to the formation of immune complexes. These complexes activate the complement system and engage Fc receptors on antigen-presenting cells like macrophages and dendritic cells. This immune activation triggers the release of pro-inflammatory cytokines which increases chronic synovial inflammation and promote joint tissue destruction in rheumatoid arthritis. In addition to their inflammatory role, ACPA have been shown to directly stimulate bone resorption [32].	Anti-CarP antibodies recognize and attach to carbamylated proteins, leading to the formation of immune complexes. These complexes can accumulate in joint tissues, where they trigger activation of the complement system. This activation attracts macrophages and neutrophils and stimulates the production of pro-inflammatory cytokines such as TNF-α and IL-6. The resulting inflammatory environment contributes to synovial membrane damage and joint destruction [33].	Mechanisms leading to the generation of AAPAs need to be characterized [34].	Anti-G6PI antibodies interact with soluble or extracellular G6PI that is released during cellular stress or injury. This binding leads to the formation of immune complexes, which activate the complement system and trigger Fc receptor engagement on immune cells. This activates neutrophils, macrophages, and increases proinflammatory cytokines, developing synovial inflammation and joint degradation [35].	Type II collagen is a key structural protein found in articular cartilage and is essential for maintaining joint integrity. In RA, the immune system target type II collagen, resulting in the production of anti-CII antibodies. These antibodies then bind to type II collagen, initiating an immune reaction that contributes to the degradation of cartilage [36].
Diagnostic value	RF is detected in approximately 70 to 80% of RA patients, especially in those with more established or severe disease [29]. RF is included in the 2010 ACR/EULAR classification criteria for RA [37]. Higher titers of RF increase the likelihood of RA, particularly in combination with clinical symptoms and other antibodies like anti-CCP [29].	ACPA can be detected years before the appearance of clinical symptoms [32]. ACPA are present in approximately 60 to 70% of individuals with RA [32]. ACPA is included in the 2010 ACR/EULAR classification criteria for RA [37].	The anti-CarP are less sensitive than other markers, but its high specificity and presence in seronegative RA enhance its diagnostic value when used in combination with clinical findings [33]. Correlated with more severe prognosis of RA [33].	AAPA can also be detected in a portion of seronegative patients for ACPA and anti-CarP, suggesting they may offer additional diagnostic insight [34].	Useful in diagnosing seronegative RA patients, where RF and ACPA are not detected [35]. Correlated with more severe prognosis of RA [35].	The anti-type II collagen has low specificity. Is found in other diseases, such as osteoarthritis and other autoimmune conditions [36]. Also is low sensitivity, is not found in the majority of RA patients seropositive for ACPA or RF [36].

**Table 2:** Studies of autoantibodies generation in animal models of RA. CIA, collagen-induced arthritis; AIA: adjuvant-induced arthritis; PIA: pristine-induced arthritis; hTNFtg: TNF transgenic mice; CAIA: collagen antibody-induced arthritis; ACPA, anti-citrullinated protein antibodies; anti-CarP, anti-carbamylated protein antibodies; RF, rheumatoid factor; anti-GPI, anti-glucose phosphoisomerase antibodies; anti-CCP, ANTI-cyclic citrullinated peptide; RA, rheumatoid arthritis; Ig, immunoglobulin.

RA Model	Animal	Auto-Antibodies	Studies
CIA	Mice	ACPA −	PAD4 expression in the synovial but anti-CCP negative [89].
			anti-CCP negative in serum [15,16].
			anti-CCP not detected in serum [91].
		ACPA +	Positive anti-CCP in serum days after boosting [88].
			Positive anti-CCP in early RA, which correlates with inflammatory state [90].
		Anti-Car-P +	Positive anti-CarP in serum (IgG1 and IgG2) in early RA stage [16].
			anti-CarP positive in serum [15].
		RF +	Positive RF IgM isotype in serum [91]
		Anti-collagen type II +	Positive anti-collagen type II (IgM isotype) at day 15 of induction [92].
			Positive anti-collagen type II (IgG isotype) at day 32 of induction [92].
	Rat	ACPA −	anti-CCP not detected in serum [92].
			anti-CCP negative in serum [16].
		Anti-Car-P +	anti-CarP positive in serum [16].
		RF −	RF not detected in serum [92].
	Rhesus monkey	ACPA −	Not detected serum CCP2-citrullinated, fibrinogen-citrullinated or myelin basic protein-citrullinated [93].
		Anti-Car-P +	Anti-CarP antibody levels detected prior to the clinical onset of RA [93].
		RF −	RF-IgM was undetectable [93].
AIA	Rat	ACPA −	anti-CCP negative in serum [16].
			anti-CCP not detected in serum [92].
		Anti-Car-P +	anti-CarP positive in serum [16].
		RF −	RF not detected in serum [92].
PIA	Rat	ACPA −	anti-CCP negative in serum [16].
		Anti-Car-P +	anti-CarP positive in serum [16].
hTNFtg	Mice	ACPA −	anti-CCP negative in serum [16].
		Anti-Car-P +	anti-CarP negative in serum [16].
CAIA	Mice	ACPA −	anti-CCP negative in serum [16].
		Anti-Car-P +	anti-CarP negative in serum [16].
K/BxN	Mice	Anti-GPI +	IgG anti-GPI autoantibodies are detected in serum (IgG1 and IgG2) [97].
			anti-GPI were found in the spleen, other lymphoid tissues and the synovial fluid [98].

## Data Availability

Not applicable.

## Abbreviations

The following abbreviations are used in this manuscript:

AAPAs	anti-acetylated protein antibodies
ACPA	anti-citrullinated protein antibodies
AIA	adjuvant-induced arthritis
ANA	anti-nuclear antibody
anti-CarP	anti-carbamylated protein antibodies
CAIA	collagen antibody-induced arthritis
CIA	collagen-induced arthritis
G6PI	glucose-6-phosphate isomerase
HLA	human leukocyte antigen
hTNFtg	TNF transgenic mice
NET	neutrophil extracellular trap
PAD	protein arginine deiminase enzymes
PIA	pristine-induced arthritis
RA	Rheumatoid arthritis
RANKL	receptor activator of nuclear factor kappa-B ligand
RF	rheumatoid factor
